# Fabrication of a highly sensitive pressure sensor based on Arabic gum polyacrylic acid nano-composite hydrogel enhanced with RGO/AgNPs

**DOI:** 10.1039/d5ra05866j

**Published:** 2025-09-15

**Authors:** Asala Saleh, Borhan Albiss

**Affiliations:** a Nanotechnology Institute, Jordan University of Science and Technology B.O.Box Irbid 3030 Jordan baalbiss@just.edu.jo AsalaSalehamsaleh20@sci.just.edu.jo

## Abstract

Stretchable and flexible pressure sensors have attracted significant interest in a wide range of applications, including smart robots and health monitoring. However, many current materials lack the needed combination of flexibility, strength, and electrical conductivity. This study aimed to develop a nanocomposite hydrogel for a pressure sensor with enhanced mechanical and electrical properties. The sensor was fabricated by incorporating silver nanoparticles (AgNPs), and reduced graphene oxide (RGO) into Arabic gum (AG) polyacrylic acid hydrogel (PAA), resulting in a more flexible and conductive polymer that features a relatively fast response time, quick recovery time, and a high sensitivity to pressure changes and human motion for high-performance sensing. The hydrogel synthesis involved *in situ* polymerization of AA and AG, followed by physical crosslinking between polymer carbonyl groups and Fe^3+^ in the presence of AgNPs and RGO, which were produced using ascorbic acid as a green reducing agent. In addition, the sensing behaviour was evaluated under different loading geometric tips—one with a square cross-section and the other shaped like a pyramid. The nanocomposite hydrogel demonstrates high sensitivity (0.136–1.832) kPa^−1^ and rapid response and recovery times (28–32 ms/62–502 ms) in a low detection range (1.15–5.77) kPa. Different techniques, including Scanning Electron Microscopy (SEM), Atomic Force Microscopy (AFM), Fourier Transform Infrared Spectroscopy (FT-IR), and X-ray Diffraction (XRD), were implemented to characterize the physicochemical properties of the nanocomposite hydrogel, while the mechanical properties of the sensor surface were investigated using nanoindentation analysis. The results showed that the nanocomposite hydrogel is a promising candidate for the next generation of flexible and wearable pressure sensing devices.

## Abstract 2

Schematic illustration of sensor performance under various mechanical deformations: response to applied pressure demonstrating the versatility and sensitivity of the hydrogel-based sensor.

## Graphical abstract

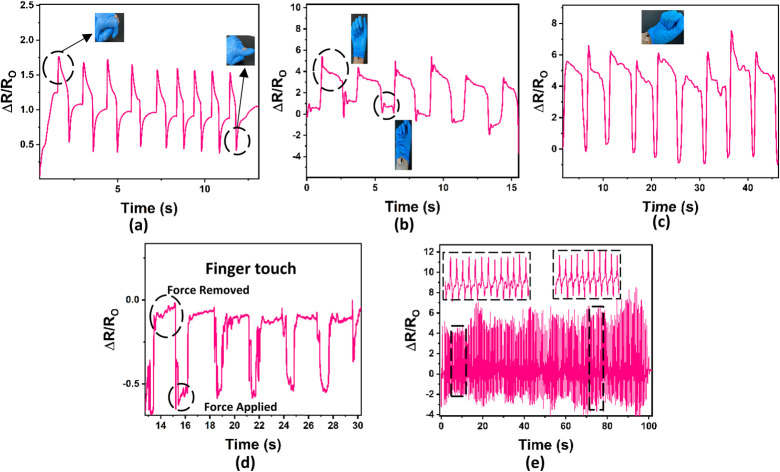

## Introduction

Flexible and stretchable sensors have attracted a lot of interest in various applications in wearable electronics, health monitoring, robots, smart textiles and energy storage. In the future, technologies that rely on flexible sensors will transform lives and provide several opportunities in industries.^1–6^ Sensors are devices that can detect and translate external stimuli from the surroundings into electrical signals.^7^ Optical, capacitive, resistive, magnetic, strain, and inductive sensors represent different kinds of sensor devices.^8^ Stretchable sensors are safer and more seamlessly interact with human skin compared to rigid sensors, they should be used in various types of electronic devices. Their good integration potential, high degree of flexibility, and lightweight are additional advantages.^9–12^ The main types of stretchable and flexible sensors are physiological stimuli and wearable electronics. Physiological stimuli are flexible devices that can detect changes in pressure, temperature, and strain and translate them into an electrical signal. On the other hand, both human machines and soft robots represent two types of wearable electronic devices. Soft conductive materials should be used to synthesize highly flexible, sensitive, stretchable, and reliable sensors, including conductive rubber, hydrogel, and leather.^5,13^ The incorporation of conducting hydrogel into the flexible substrate shows a high improvement in sensor efficiency.^14^

The similarity in mechanical properties between hydrogel and organic tissue will lead to a better connection between human skin and stretchable hydrogel. Therefore, the resistance and accuracy of capacitance for the hydrogel sensor will increase. In addition, the high biocompatibility and availability of natural polymers make them more attractive than synthetic polymers in the preparation methods of the sensor.^15^ Low conductivity of the hydrogel makes it unsuitable for flexible sensor fabrication, as a result, additives like nanowires, carbon nanotubes, graphene, graphene oxide (GO), nanoparticles, and metal salt help to overcome its conductivity issue, enhance tensile strength, and reduce their rigid nature. Also, using tough-type hydrogel is critical for skin device synthesis.^1,13,15,16^ Arabic gum (AG) is a neutral polysaccharide extracted from the acacia tree. The availability, biodegradability, biocompatibility, good antimicrobial properties,^17^ and solubility of AG in water make it a suitable choice for several applications, including sensors, batteries, and film formation.^18,19^ Polyacrylic acid (PAA) is an anionic organic polymer. The presence of a carboxyl group (COOH) in the hydrogel matrix enhances its bonding to different surfaces, which facilities the free radical polymerization reaction between acrylic acid and arabic gum in the presence of Fe^3+^ to enhance hydrogel properties.^20–22^

The high surface area, low toxicity, optical properties, low cost, and remarkable biological, chemical, and physical properties and small size of silver nanoparticles (AgNPs) make them a suitable choice for various applications, especially in sensors. To prevent AgNP agglomeration and stabilize its growth. GO have been added to hydrogel matrix during the polymerization reaction.^23–26^ Graphene and its derivatives are two-dimensional materials with high conductivity, excellent mechanical and electrical properties, biocompatibility, and high surface area, which make it ideal material for fabricating flexible sensors.^10,27^ In this study, the developed nanocomposite hydrogel is composed of PAA, AG, AgNPs, and reduced graphene oxide (RGO). This resulted combination of natural polymer, synthetic polymer, and nanomaterials is designed to overcome the low conductivity and poor toughness of hydrogel. The resulting nanocomposite hydrogel exhibits good flexibility and sensitivity, making it a suitable material for pressure sensors and human motion detection, and shows strong potential for several wearable sensor applications.

## Experimental methods

### Materials

Iron(iii) chloride (FeCl_3_·6H_2_O), ascorbic acid (VC), Arabic gum (AG), acrylic acid (AA), silver nitrate (AgNO_3_), potassium per manganite (KMNO_4_), hydrogen peroxide (H_2_O_2_), graphite powder, nitric acid (HNO_3_), sulfuric acid (H_2_SO_4_), hydrochloric acid (HCl), and ammonium per sulfate (APS) were all purchased from Sigma-Aldrich. All chemicals were utilized as given without purification. Dilution and standard solutions were prepared using deionized water following standard procedures.

### Synthesis of nanocomposite hydrogel

**Fig. 1:**
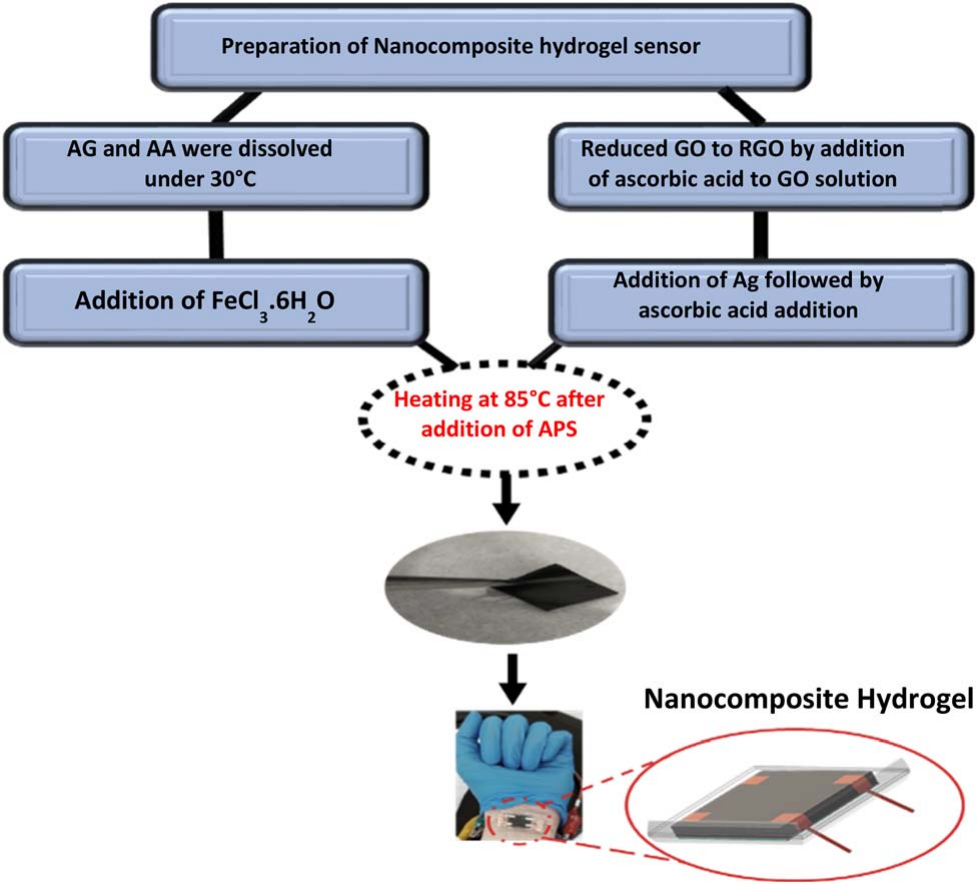
Schematic diagram of the preparation process of nanocomposite hydrogel. 37.5 (wt/wt)% of Arabic gum and 62.5 (wt/wt)% of acrylic acid were first mixed to form a uniform solution. Silver nanoparticles were synthesized *in situ* using a green reducing agent (ascorbic acid), while reduced graphene oxide was added into the polymer matrix to enhance conductivity. The mixture went through an *in situ* polymerization, followed by physical crosslinking with Fe^3+^ ions was employed to create a stable hydrogel network. The final nanocomposite hydrogel was shaped into the sensor shape and employed for pressure sensing applications.

Hummers' method was used to synthesize graphene oxide (GO).^28^ First, 2.5 g of sodium nitrate was mixed with 120 ml of sulfuric acid and stirred for 10 minutes. Next, 4.5 g of graphite powder was added at 0 °C, followed by the slow addition of 15 g of potassium permanganate to the solution. This mixture was stirred for 2 hours at 40 °C using an overhead stirrer. After that, at a (4–0) °C temperature range, 250 ml of deionized water (DI) was added slowly, followed by the addition of 20 ml of hydrogen peroxide into the mixing solutions. The resulting GO was washed three times with 10% hydrochloric acid and three times with deionized water. The washed GO solution was then separated by centrifuge and left at RT to dry slowly. For the preparation of nanocomposite hydrogels, the free radical polymerization reaction was utilized based on our previous work. A conductive hydrogel was prepared by mixing a neutral hydrogel matrix of AG and acrylic acid with conductive agents, including GO and Ag metal salt. During the polymerization reaction, ascorbic acid, as a reducing agent was used to reduce GO to RGO and produce nanoparticles from silver metal. This physically cross-linked hydrogel was prepared by dissolving 1.2 g of AG in 40 ml deionized water and then adding 2 g of acrylic acid at 30 °C. Subsequently, 0.087 g of FeCl_3_·6H_2_O was incorporated into the homogeneous solution. Which was stir at RT for 3 h (flask A). In another flask (flask B), 0.04 g of GO was dissolved in 5 ml of deionized water using ultrasonication for 1 h. Afterward, 100 mg of ascorbic acid was added slowly, after stirring for 30 minutes, 1 ml of Ag solution (120 mg/4 ml) was added dropwise, followed by an additional 100 mg of ascorbic acid. After another 30 minutes, flask A was added slowly to flask B while stirring contentiously. The initiator APS (0.05 g) was added to the solution and allowed to mix for 3 h at 85 °C. Finally, the nanocomposite hydrogel was allowed to dry at RT for three days.^29^Fig. 1 shows the schematic diagram of the nanocomposite hydrogel preparation.

### Fabrication of a strain sensor

**Fig. 2:**
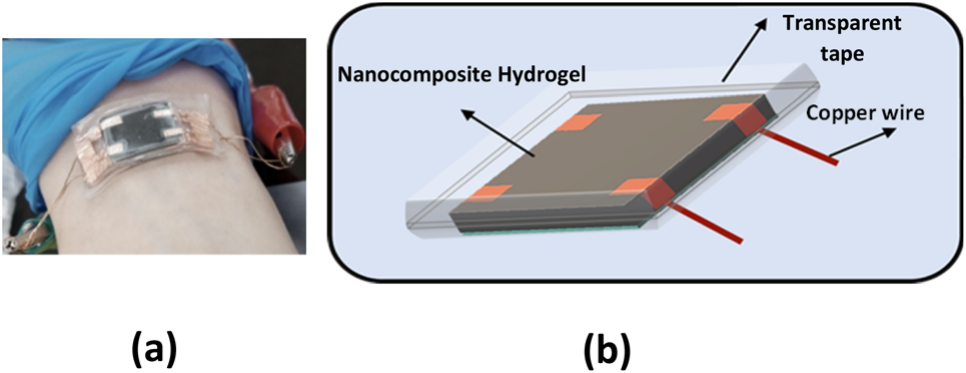
(a) Photograph of the flexible nanocomposite hydrogel pressure sensor. (b) Schematic diagram of nanocomposite hydrogel.

Fig. 2(a) shows the nanocomposite hydrogel strain sensor. The schematic diagram of the nanocomposite hydrogel flexible pressure sensor is shown in Fig. 2(b). The fabricated sensor consists of a nanocomposite-hydrogel connected to copper tape. The high flexibility of hydrogel makes it suitable to be use in wearable electronic device applications.

## Results and discussion

### Morphology study of nanocomposite hydrogel

**Fig. 3:**
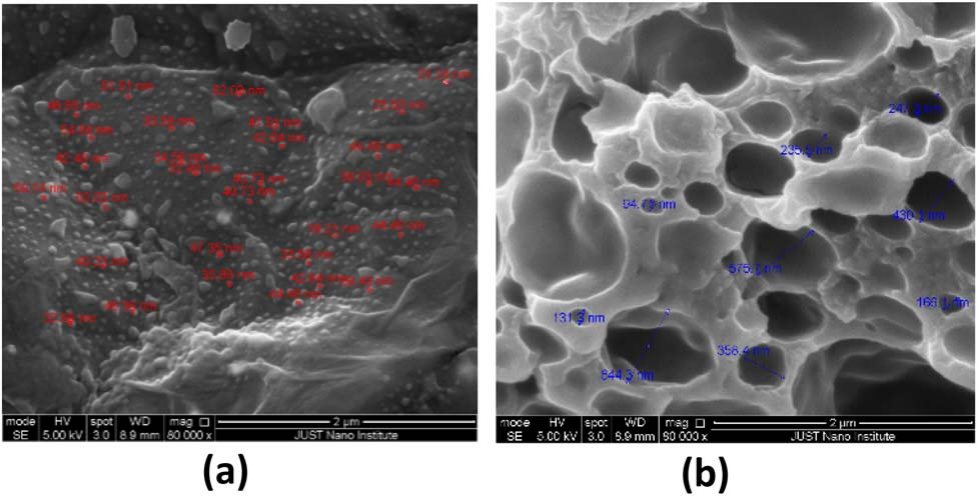
SEM images for nanocomposite hydrogel: (a) the distribution of AgNPs on the surface of nanocomposite hydrogel across the hydrogel surface indicating successful interaction with polymer surface, and (b) the highly porous network structure of nanocomposite hydrogel that facilitates sensitivity and response efficiency.

The SEM method is a specific tool for studying hydrogel morphology and its three-dimensional structure.^30^Fig. 3 (a and b) shows SEM images of the nanocomposite Arabic gum hydrogel. The distribution of small-sized AgNPs with an average pore diameter of 20–50 nm on the surface of the hydrogel is shown in Fig. 3(a). While, Fig. 3(b) shows the high porosity nature of the prepared Arabic gum nanocomposite hydrogel with an average pore diameter of 130–600 nm. The higher porosity density of nanocomposite hydrogel plays a significant role in hydrogel sensitivity toward external stimuli like pressure, strain, and external sound.

**Fig. 4:**
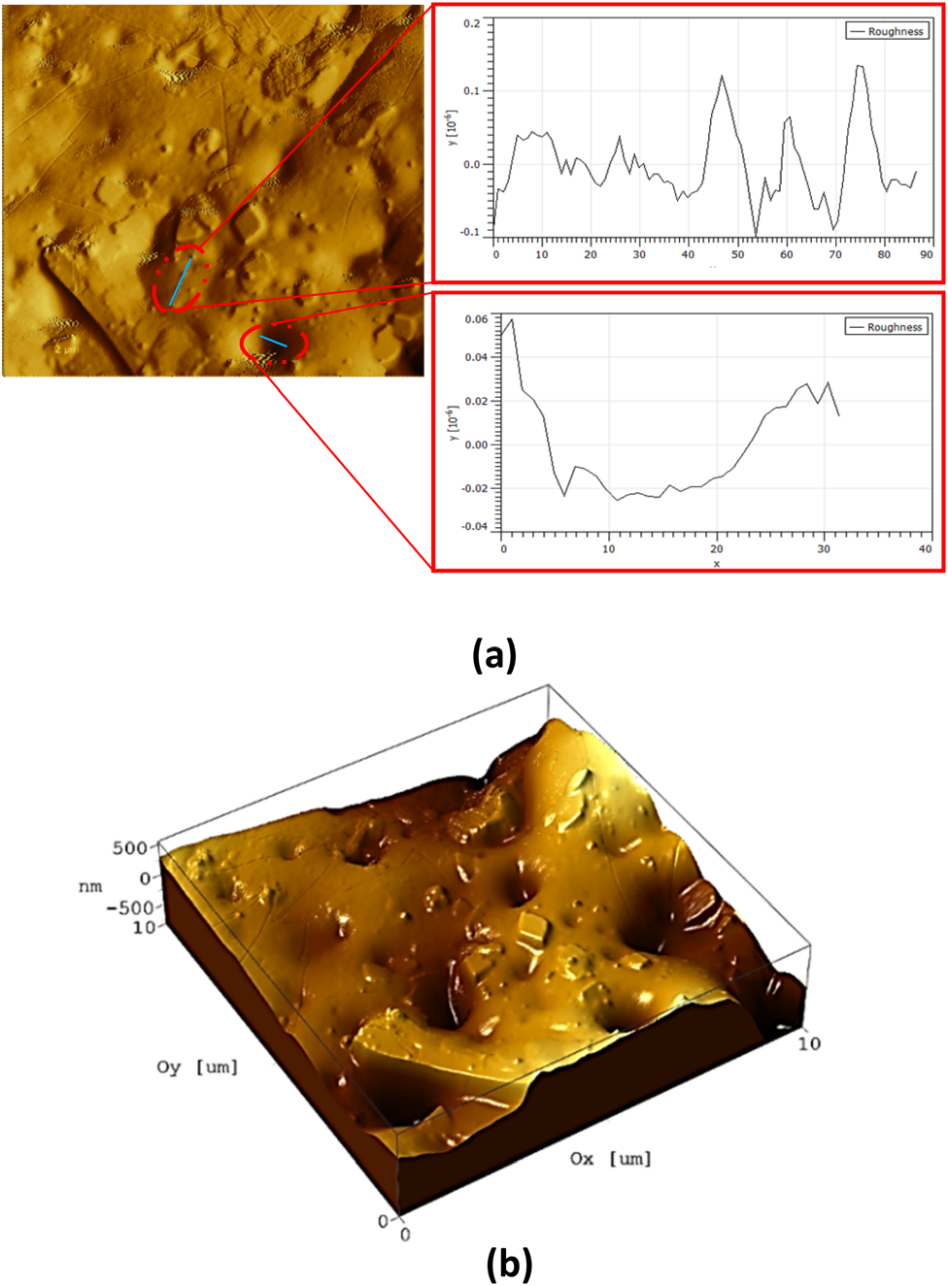
AFM analysis of the nanocomposite Arabic gum hydrogel: (a) topographical and phase images of the nanocomposite Arabic gum hydrogel, and (b) three-dimensional AFM images of nanocomposite hydrogel indicate the porosity and the presence of small size of AgNPs embedded within surface of the hydrogel matrix.

Furthermore, the presence of AgNPs will enhance the conductivity and stability of the pressure sensor device.^31^ The topography of the nanocomposite hydrogel was studied using AFM methods. AFM characterization is an important technique for studying hydrogel surface roughness.^32^ The topographical feature of nanocomposite hydrogel reveals information about the size and distribution of AgNPs, as well as the porosity density on the hydrogel surface. Fig. 4(a) shows the topographic and phase images of the nanocomposite hydrogel. The dark region on the figure refers to the pores while the small white circle refers to the AgNP sphere. Fig. 4(b) shows the three-dimensional images of the nanocomposite hydrogel, where the pores and AgNPs increase the roughness of the surface with an average value of roughness (*R*_a_) of 0.197225 μm and an RMS roughness (*R*_q_) of 0.275 μm.

### FTIR and XRD

**Fig. 5:**
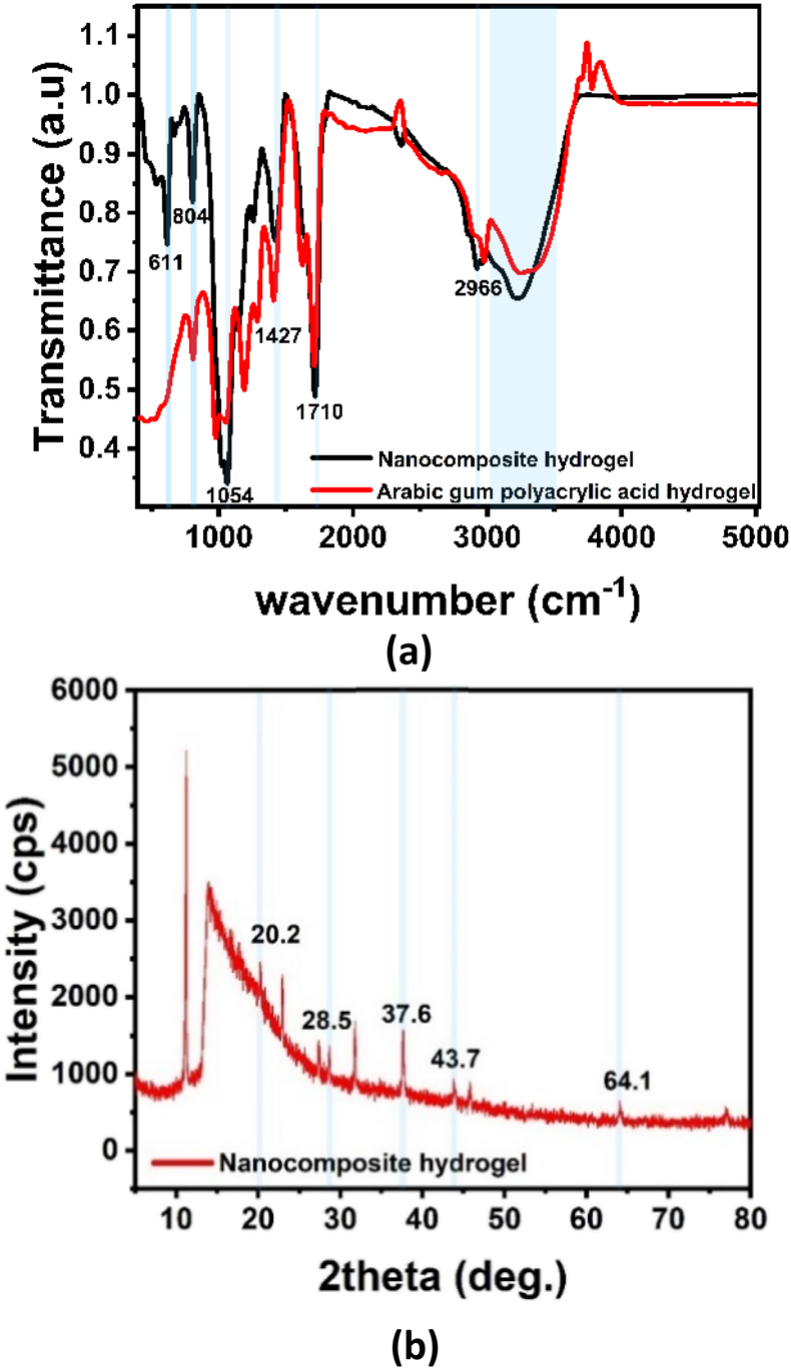
Structural characterization of the nanocomposite hydrogel: (a) FTIR spectra of arabic gum polyacrylic acid hydrogel (red) and nanocomposite hydrogel (black), and (b) XRD pattern conforming the AgNPs crystalline structure.

FTIR spectroscopy was performed to confirm the successful polymerization reaction and formation of AGPAA hydrogel and investigate the interaction between RGO/AgNPs and polymer matrix. Fig. 5(a) shows the FTIR analysis of the nanocomposite hydrogel strain sensor. The peaks indicate the formation of AGPAAc, where the absence of peaks at 2500 cm^−1^, which represent C=C stretching vibration, refers to the effect of using APS as an initiator in the polymerization reaction of acrylic acid AA. While the peak at 1710 cm^−1^ refers to C=O stretching vibration in carbonyl groups, which are found in RGO and AGPAAc hydrogel. Peaks at 804 cm^−1^ and 1054 cm^−1^ refer to the stretching vibration of C–O–C and C–O–H in AG, respectively. The peaks at 1427 cm^−1^ and 2917 cm^−1^ refer to the pending vibration of CH_2_ and the stretching vibration of C–H sp^3^ (symmetric) in the resulting hydrogel, respectively. The AgNP effect appears in the peak at 611 cm^−1^, which indicates the Ag–O bond. The range from 3150–3600 cm^−1^ shows a sharper peak on –OH stretching vibration of nanocomposite hydrogel compared with a broad peak in Arabic gum polyacrylic acid hydrogel, resulting from multiple electrostatic interactions between the oxygen functional group on AGPAA hydrogel, RGO, and Ag–O bond with Fe^3+^ ions in the hydrogel matrix.

While FTIR confirm the chemical interaction, the XRD analysis used for surface identification and crystalline structure of nanoparticles incorporation in the polymer matrix. Fig. 5(b) shows the XRD analysis of the prepared nanocomposite hydrogel. The AG polymer shows two peaks at 20.8° and 28.5°, indicating its amorphous phase in the hydrogel matrix. The three peaks at (37.6°, 43.7°, and 64.1°) are the three main peaks of AgNPs that refer to their crystalline structure. These peaks correspond to crystallographic planes at (111, 200, and 220), respectively. Furthermore, the 001 pattern reflection intensity of GO at approximately 11° decreased in the polymer composite from 40 000 (used GO) to 5000 in the composite hydrogel, representing an approximately 87.5% reduction in peak intensity. This significant decrease, along with the presence of a broad peak between 15–20°, aligns with the reduction of GO and the formation of RGO.

### Nanoindentation test

**Fig. 6:**
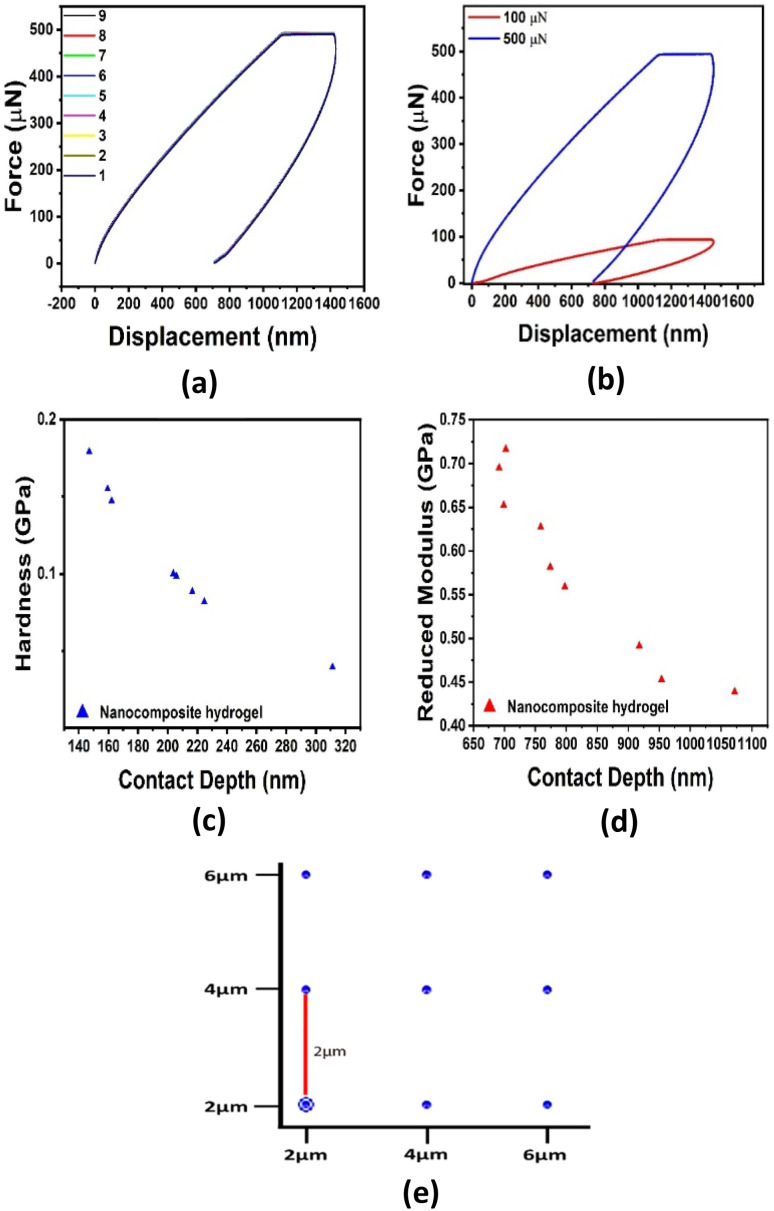
Nanoindentation test for nanocomposite hydrogel at (a) 500 μN, (b) two different applied forces at 100 and 500 μN, (c) hardness plot for 9-point on the hydrogel surface, (d) reduced modulus of nanocomposite hydrogel, (e) nine-points array method to study the uniform mechanical properties of hydrogel samples.

The nanoindentation method was used to study the mechanical properties of nanocomposite hydrogel, which provides information about the hardness and elastic modulus of the dried hydrogel matrix.^33^ The array patterns were fixed for the hydrogel to be 9 points with the same space distance (2 μm) and fixed applied force as shown in Fig. 6(e). Fig. 6(a) shows the nanoindentation test for nanocomposite hydrogel when the applied force was 500 μN. Fig. 6(b) shows the nanoindentation curve for nanocomposite hydrogel at 500 and 100 μN applied forces, a large difference in curve shape indicating a change in the mechanical properties of samples with increasing applied force. Fig. 6(c) and (d) show the hardness curve and the elastic modulus change for the nanocomposite hydrogel at nine different points taken on the hydrogel surface, respectively.

### Pressure sensor test

**Fig. 7:**
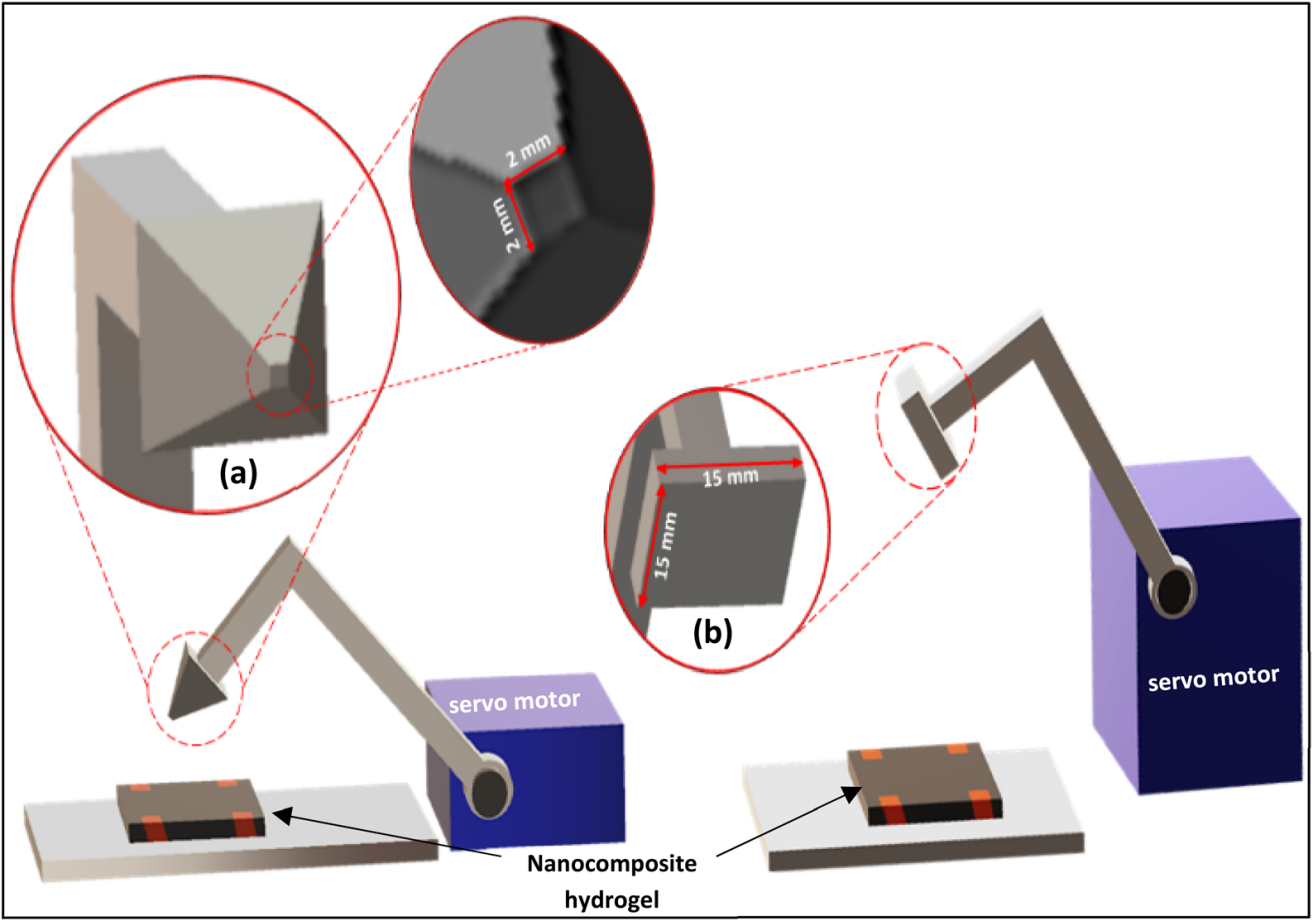
3D-model of the force sensor based on probe tapper (a) pyramid end, and (b) square end used to produce a specific pressure on the surface of the nanocomposite hydrogel sensor.

Fig. 7 shows a 3D model of the device used to study the force-sensing efficiency of the nanocomposite hydrogel. As shown in the figure, the setup consisted of a servo motor attached to a probe tapper with either a pyramid end (Fig. 7(a)) or a square end (Fig. 7(b)). When these geometries touch the nanocomposite hydrogel's surface, the applied force will translate to an electrical resistance change, which indicates the sensor's response to different geometries. The pyramid end area is (2 mm × 2 mm), where the square end area is (15 mm × 15 mm).

**Fig. 8:**
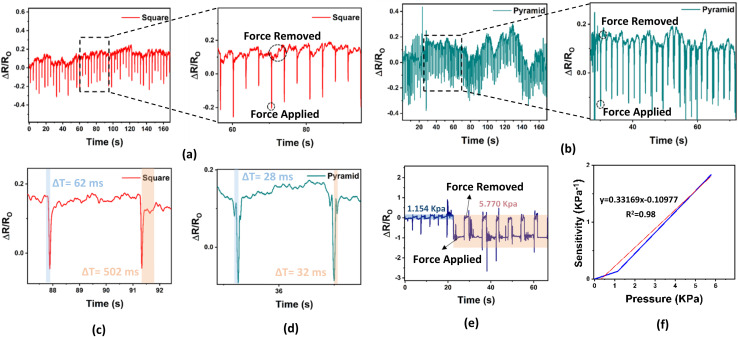
Schematic diagram of pressure sensor versace time produced by (a) square and (b) pyramid tapper end, response time and release time of the nanocomposite hydrogel sensor using probe tapper (c) square and (d) pyramid tips. (e) Relative resistance changes with compression of 1.154 kPa and 5.770 kPa of pressure. (f) Sensitivity of the nanocomposite hydrogel sensor when different pressures (1.154 kPa and 5.770 kPa) were applied.

Fig. 8 shows a schematic diagram of the relative resistance change (Δ*R*/*R*_O_) with respect to time in seconds. When the geometrics tip (pyramid or square tips) contacts with nanocomposite hydrogel surface, the change in Δ*R*/*R*_O_ decreases, and when the applied force is removed, the Δ*R*/*R*_O_ returns to its baseline. Fig. 8(a and b) shows the relation between geometric tips and the value of Δ*R*/*R*_O_; the change in resistance and response time will depend on the contact area, pressure distribution, and the concentrated point of the geometric tips. The pyramid-shaped tip has a small contact area with lower pressure distribution compared with a square-shaped tip, which leads to a faster pressure change on the surface of the nanocomposite hydrogel when the pyramid tip touches it, resulting in a high resistance change on the hydrogel. The nanocomposite hydrogel sensor was very sensitive to how the force was applied on its surface, which means the contact geometry can significantly influence nanocomposite hydrogel behavior. The response time of the nanocomposite hydrogel sensor was measured by applying fixed force and then recording the time at which the Δ*R*/*R*_O_ of the sensor reach 90% of its maximum value, while the recovery time was determined by removing the applied force and recording the time at which the Δ*R*/*R*_O_ of the sensor began to return to the baseline by using either a square or pyramid tips. Fig. 8(c) shows a response and recovery time of the sensing hydrogel when a square tip was used; the Δ*R*/*R*_O_ dropped from 0.1652 to approximately −0.042 within a 62 ms response time, and returned to 10% of its baseline within 502 ms recovery time. The square tip with more contact area distributes the pressure over a larger area, leading to lower recovery as the hydrogel sensor needs more time to return to its original shape. Fig. 8(d) shows the response and recovery time of the sensing hydrogel when the pyramid tip is used, the Δ*R*/*R*_O_ dropped from 0.1091 to approximately −0.0925 within 28 ms response time, and return to 10% of its baseline within 32 ms recovery time, which shows faster response/recovery time compared with (PVA- and MXene, LBG, CNTs, and PVA, PVA/AgNP hydrogel) and comparable to the CNT- and Fe^3+^ cross-linked hydrogels as reported in Table 1.

**Table 1:** Summary and conversion of the latest pressure sensor

Sensor	Reported sensitivity	Pressure range	Response/Recovery	Ref.
AGPAA/GO/AgNPs	0.13581–1.8315 kPa^−1^	1.15–5.77 kPa	(28–32) ms and (62–502) ms	This work
PVA/MXene composite-based sensor	0.45 kPa^−1^	2–10 kPa	500 ms/500 ms	34
LBG, CNTs, and PVA	12.7 kPa^−1^	<20 kPa	263 ms/315 ms	35
OVA/PAM/Fe^3+^	2.9 kPa^−1^		18 ms/19 ms	36
PVA/AgNP hydrogel	0.017 kPa^−1^	0–22 kPa	758 ms/536 ms	37
GO/PPy@PU (polyurethan)	0.79 kPa^−1^	Less than 2.5 kPa	<70 ms	38

The rapid response and recovery time with very close value, result from focused pressure on very small area. As a result, the deformation ability of the hydrogel was much higher when force was applied in a focused way. Fig. 8(e) shows the relation between Δ*R*/*R*_O_ and pressure. At first, the blue covered area indicates the Δ*R*/*R*_O_ value when a 1.154 kPa was applied, followed by a large change in Δ*R*/*R*_O_ value when pressure increased to 5.770 kPa (pink covered area). The sensitivity of the nanocomposite hydrogel increased from 0.13581 kPa^−1^ to 1.8315 kPa^−1^ when the pressure increased from 1.1541 kPa to 5.77 kPa, as shown in Fig. 8(f). The sensitivity (*S*) of the pressure sensor was calculated using the equation:1
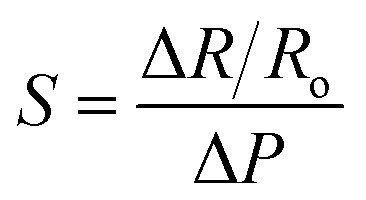
where *R*_o_ is the initial resistance, Δ*R* is the resistance change (*R*–*R*_o_), and Δ*P* is the pressure change (*P*–*P*_o_). The increase in pressure from (1.15–5.77) kPa shows a linear response with *R*^2^ = 0.98. Therefore, the hydrogel sensor isn't only capable of force detection but also translates the force changes into Δ*R*/*R*_O_ shifts, as a result, the table highlights that our pressure sensor shows a promising balance between sensitivity, pressure detection range, and response to recovery time, indicating its strong potential for various sensing applications.

### Human motion sensor device test

**Fig. 9:**
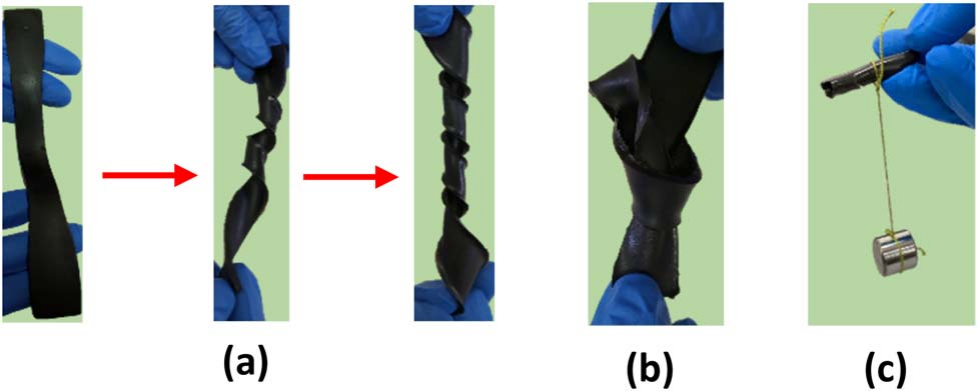
Mechanical properties of the hydrogel (a) knotting (b) twisting, and (c) a dried sample loads 300 g weight.

Fig. 9(a and b) shows the highly flexible structure of the nanocomposite hydrogel. The prepared hydrogel can handle repeated tension, crimping, knotting, and bending without cracking or tearing, indicating its high flexibility and elasticity. The high rigidity of the hydrogel increased its ability to hold a piece of metal without breaking (Fig. 9(c)).

**Fig. 10:**
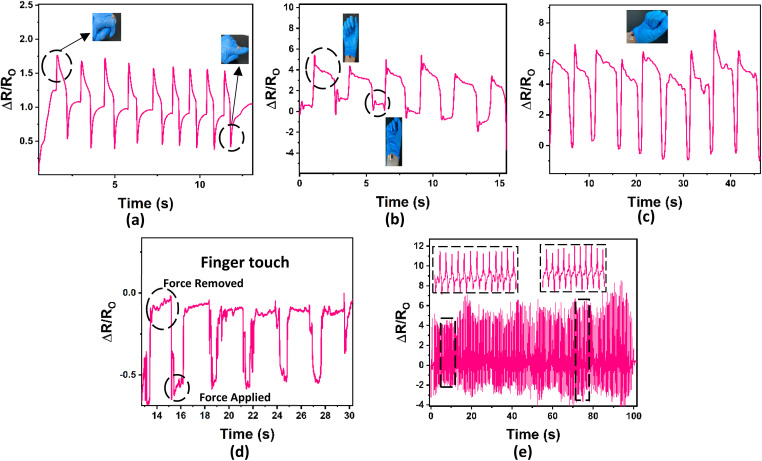
Schematic illustration of sensor performance under various mechanical deformations: (a) bending from 0% to 90%, (b and c) wrist bending from 0% to 90% at different holding times, (d) finger touch interaction, and (e) response to applied pressure demonstrating the versatility and sensitivity of the hydrogel-based sensor.

Fig. 10 shows the ability of the nanocomposite hydrogel to detect different external stimuli. The effect of the bending angle of the finger and wrist was studied by fixing the nanocomposite hydrogel on the human skin (Fig. 10(a–c)). Fig. 10(a) shows the Δ*R*/*R*_O_ after finger bending, where the Δ*R*/*R*_O_ value was increased to its maximum when the bending increased from 0% to 90%. Fig. 10(b) shows the nanocomposite hydrogel sensor bending, in which the sensor was fixed on the wrist, and the hand was slowly raised, causing a Δ*R*/*R*_O_ change. The bending of the wrist was also tested at a 5 seconds holding time (Fig. 10(c)). The nanocomposite hydrogel sensor sensitivity toward pressure using a human finger was also considered (Fig. 10(d)), where the finger was pressed on the sample several times with noticeable Δ*R*/*R*_O_ change. These results show a high recovery of hydrogel after five cycles. Fig. 10(e) shows the air pressure response when a fixed amount of air was applied to the sensor surface, resulting in a constant Δ*R*/*R*_O_ change and high sensitivity of the hydrogel; the figure shows the high recovery of the hydrogel after more than 100 cycles. This trend indicates that the hydrogel's sensitivity to large-scale human motion and air pressure changes will enhance its performance in detecting variations in environmental stimuli and conditions.

## Conclusions

In this study, a nanocomposite hydrogel based on Arabic gum polyacrylic acid was successfully prepared *via* free radical polymerization reaction where –COO– groups of polymeric chains physically cross-linked with Fe^3+^, followed by RGO/AgNPs addition as a conductive material, resulting in a flexible, highly sensitive nanocomposite hydrogel that can handle repeated crimping, knotting, and bending without tearing. The addition of RGO and AgNPs was crucial for enhancing the mechanical and electrical properties of the nanocomposite hydrogel sensor, making it suitable for use in pressure sensing applications. The fabricated sensor shows different response behavior when different geometric tips are used to apply force to its surface, indicating the effect of contact area and pressure disruption on sensor sensitivity and Δ*R*/*R*_O_ change. This new nanocomposite material will open the door for highly sensitive monitoring and sensing devices. Additionally, the high sensitivity of nanocomposite hydrogel was utilized to develop a sensor that has the efficiency to detect both human motion, force with varied geometries, and air pressure. The sensor exhibited high sensitivity (0.136–1.832) kPa^−1^ within a low detection range (1.15–5.77) kPa and fast response and recovery times (28–32 ms/62–502 ms respectively), as well as highly sensitive properties to any external stimuli. Overall, the nanocomposite hydrogels' combination of pressure and human sensing makes them a promising, adaptable tool for both environmental and electronic applications.

## Author contributions

The authors confirm their contribution to the paper as follows: study conception and design: Borhan Albiss, Asala Saleh; data collection: Asala Saleh; analysis and interpretation of results: Borhan Albiss, Asala Saleh; draft manuscript preparation: Borhan Albiss, Asala Saleh. All authors reviewed the results and approved the final version of the manuscript.

## Conflicts of interest

There are no conflicts to declare.

## Data Availability

The paper and its SI contain the data that back up the study's findings. On reasonable request, further information can be obtained from the corresponding author.
